# The Truncated C-terminal Fragment of Mutant ATXN3 Disrupts Mitochondria Dynamics in Spinocerebellar Ataxia Type 3 Models

**DOI:** 10.3389/fnmol.2017.00196

**Published:** 2017-06-20

**Authors:** Jung-Yu Hsu, Yu-Ling Jhang, Pei-Hsun Cheng, Yu-Fan Chang, Su-Han Mao, Han-In Yang, Chia-Wei Lin, Chuan-Mu Chen, Shang-Hsun Yang

**Affiliations:** ^1^Institute of Basic Medical Sciences, College of Medicine, National Cheng Kung UniversityTainan, Taiwan; ^2^Department of Cell Biology and Anatomy, College of Medicine, National Cheng Kung UniversityTainan, Taiwan; ^3^Department of Physiology, College of Medicine, National Cheng Kung UniversityTainan, Taiwan; ^4^Department of Life Sciences, Agricultural Biotechnology Center, National Chung Hsing UniversityTaichung, Taiwan

**Keywords:** truncated aTXN3, spinocerebellar ataxia type 3, mitochondria dynamics, transgenic mice, neurodegeneration, fusion and fission

## Abstract

Spinocerebellar ataxia type 3 (SCA3), known as Machado-Joseph disease, is an autosomal dominant disease caused by an abnormal expansion of polyglutamine in *ATXN3* gene, leading to neurodegeneration in SCA3 patients. Similar to other neurodegenerative diseases, the dysfunction of mitochondria is observed to cause neuronal death in SCA3 patients. Based on previous studies, proteolytic cleavage of mutant ATXN3 is found to produce truncated C-terminal fragments in SCA3 models. However, whether these truncated mutant fragments disturb mitochondrial functions and result in pathological death is still unclear. Here, we used neuroblastoma cell and transgenic mouse models to examine the effects of truncated mutant ATXN3 on mitochondria functions. In different models, we observed truncated mutant ATXN3 accelerated the formation of aggregates, which translocated into the nucleus to form intranuclear aggregates. In addition, truncated mutant ATXN3 caused more mitochondrial fission, and decreased the expression of mitochondrial fusion markers, including Mfn-1 and Mfn-2. Furthermore, truncated mutant ATXN3 decreased the mitochondrial membrane potential, increased reactive oxygen species and finally increased cell death rate. In transgenic mouse models, truncated mutant ATXN3 also led to more mitochondrial dysfunction, neurodegeneration and cell death in the cerebellums. This study supports the toxic fragment hypothesis in SCA3, and also provides evidence that truncated mutant ATXN3 is severer than full-length mutant one *in vitro* and *in vivo*.

## Introduction

Spinocerebellar ataxia type 3 (SCA3), known as Machado-Joseph disease, is one of nine polyglutamine (polyQ) diseases that occurs due to an abnormal expansion of polyglutamine in exon 10 of the *ATXN3* gene (Kawaguchi et al., 1994). The mutant ATXN3 leads to a toxic gain in function, and results in neuropathological characteristics, such as neuronal aggregates, neurodegeneration and neuronal death, in the central nervous system (Paulson et al., 1997; Riess et al., 2008). With the progression of this disease, patients display symptoms of motor ataxia and deficit due to cerebellar and brainstem dysfunction, and also show impairment in cognitive functions (Paulson, 2012). To date, there is no cure for this disease, and clinical treatments only alleviate its symptoms in patients.

The molecular weight of the disease-causing protein, ATXN3, is approximately 42 kDa, and the exact size is dependent on the repeats of polyQ in the C-terminal region (Kawaguchi et al., 1994). The proteolytic cleavage of ATXN3 to remove the N-terminus is critical for the progression of this disease, which is known as the toxic fragment hypothesis. The truncated C-terminal fragments of ATXN3 were observed in the brains of SCA3 patients, especially in intranuclear aggregates. The truncated and expanded C-terminal fragments not only result in conformational changes to the protein, but also facilitate the formation of pathological aggregates, such as intranuclear aggregates (Jana and Nukina, 2004; Haacke et al., 2006; Simoes et al., 2012; Matos et al., 2016). Furthermore, previous studies also have shown truncated C-terminal fragments of ATXN3 are highly related to calpain-dependent proteolytic cleavage (Simoes et al., 2012; Matos et al., 2016), suggesting critical roles of ATXN3 proteolysis during the progression of SCA3. In neurons, normal ATXN3 has been confirmed to play important roles in neuronal differentiation, morphology, proliferation and survival, and also affects the ubiquitin-proteasome system, cytoskeleton construction, neuronal transmission and mitochondrial functions at the cellular level (Chou et al., 2006, 2014; Chen et al., 2014; Neves-Carvalho et al., 2015; Teixeira-Castro et al., 2015). This suggests that mutant ATXN3 causes multiple cellular dysfunctions, and then results in neuropathological characteristics and symptoms in SCA3 patients.

Mitochondrial deficit is often observed in neurodegenerative diseases, such as Parkinson’s and Huntington’s diseases. In SCA3, full-length mutant ATXN3 has been shown to increase mitochondrial-mediated cell death in different models (Tsai et al., 2004; Chou et al., 2006). In addition, full-length mutant ATXN3 is also reported to decrease mitochondrial DNA copy numbers in cell and transgenic mouse models (Yu et al., 2009; Kazachkova et al., 2013). In SCA3 patients, disturbance of mitochondrial copy numbers and complex II has also been reported (Yu et al., 2009; Laco et al., 2012). These results support the idea that full-length mutant ATXN3 exacerbates mitochondrial functions in neurodegenerative SCA3.

Since the proteolytic cleavage of ATXN3 is an important progression to generate truncated mutant C-terminal fragments and facilitate the formation of cellular aggregates (Haacke et al., 2006), whether this truncated mutant ATXN3 leads to the dysfunctions of mitochondria to support the toxic fragment hypothesis is still unknown. In this study, we used *in vitro* neuroblastoma cells and *in vivo* transgenic mouse models to examine the effects of truncated mutant ATXN3 on mitochondrial functions, and showed that truncated mutant ATXN3 exacerbated above functions, further leading to neurodegeneration.

## Materials and Methods

### DNA Construction

**FIGURE 1:**
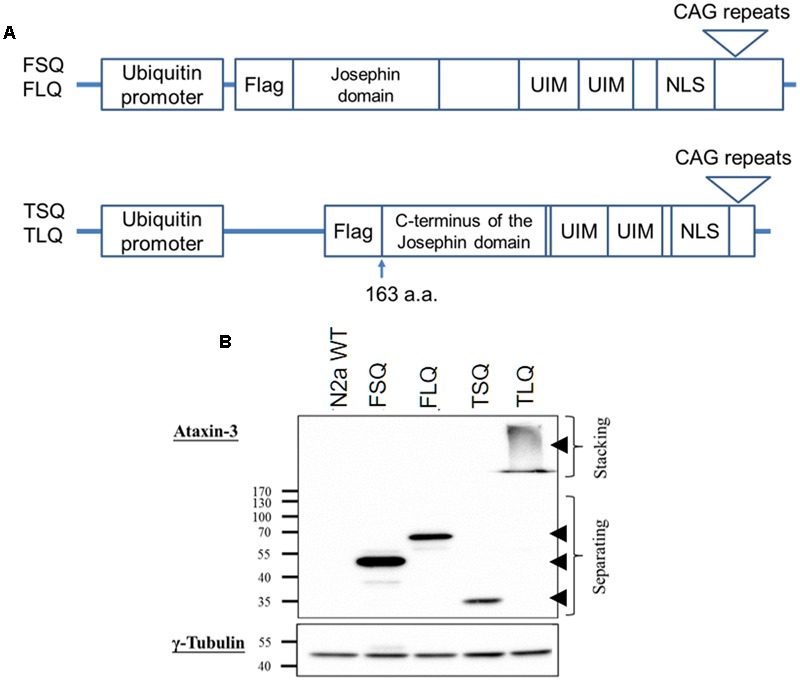
The characterization of different ATXN3 constructs. **(A)** Different ATXN3 constructs used in this study are graphed. The detailed information is described in “Materials and Methods.” UIM, ubiquitin interacting motifs; NLS, nuclear localization sequence. **(B)** N2a cells were transfected with different constructs for 48 h, and the expression of exogenous ATXN3 was detected by Western blotting using an ataxin-3 antibody, as indicated by arrow heads. The γ-tubulin was used as an internal control.

Full-length *ATXN3* cDNA with short 22 (FSQ) or long 88 (FLQ) CAG repeats in exon 10 was amplified from the ATXN3 templates gifted from Dr. Xiao-Jiang Li at Emory University based on the sequence of *ATXN3* (accession number: BC033711). The truncated forms of ATXN3 cDNA, which start from the 163rd amino acid and contain C-terminal fragment of ATXN3 with short CAG repeats (TSQ) or long CAG repeats in exon 10 (TLQ), were amplified from FSQ and FLQ, respectively. The truncated ATXN3 includes C-terminus of the Josephin domain, ubiquitin interacting motifs and nuclear localization sequence (Haacke et al., 2006). These different ATXN3 cDNAs were tagged with one Flag epitope sequence (**Figure 1**), and inserted into a lentiviral vector (Addgene plasmid: #14883), which is under the control of a human ubiquitin promoter.

### Cell Culture and Transfection

Neuron 2A (N2a) mouse neuroblastoma cells were maintained in Eagle’s minimum essential medium (MEM; Invitrogen) with 1 mM sodium pyruvate (Sigma), 10% (v/v) heat-inactivated fetal bovine serum (Hyclone), 100 U/mL penicillin and 100 μg/mL streptomycin (Invitrogen) at 37°C in a 5% CO_2_ incubator. Human SH-SY5Y neuroblastoma cells were cultured in high glucose Dulbecco’s modified Eagle’s medium (DMEM) with 2 mM L-glutamine, 1 mM sodium pyruvate and 15% fetal bovine serum (Hyclone) at 37°C in a 5% CO2 incubator. These cells were passaged every 3–5 days depending on cell confluency. To perform transfection, N2a or SH-SY5Y cells were seeded 1 day before transfection, and then plasmid DNAs, including FSQ, FLQ, TSQ, TLQ or pDsRed2-Mito Vector (Clontech), were transfected using Lipofectamine^TM^ 2000 (Invitrogen). The transfection efficiency was approximately 50–60%. Cells were kept culturing for 12–48 h as needed and then subjected to the following assays.

### Generation of Transgenic Mice

TSQ and TLQ constructs were used to generate high-titer lentiviruses for production of transgenic mice, as described before (Cheng et al., 2016). This study was carried out in accordance with the recommendations of Institutional Animal Care and Use Committee at National Cheng Kung University, Taiwan. The protocol was approved by the Institutional Animal Care and Use Committee at National Cheng Kung University, Taiwan. All methods were performed in accordance with the relevant guidelines and regulations. TSQ and TLQ constructs used for generating transgenic mice were described in the “DNA construction” part. FVB female mice were used to collect zygotes at two pronuclei stage, and then these zygotes were subjected to lentiviral transgenesis. Live pups from two constructs were genotyped using PCR primers, including Forward-primer: GAGGCGTCAGTTTCTTTGGTC located in the ubiquitin promoter region and Reverse-primer: AGTAGGCTTCTCGTCTCTTCC located in the *ATXN3* gene to detect the 654 bp amplicon. Transgenic and non-transgenic mice were used for further studies.

### Nuclear and Cytoplasmic Protein Extraction

We performed nuclear and cytoplasmic protein extraction based on a previous study (Suzuki et al., 2010). Briefly, the transfected N2a cells were cultured for 2 days, and then collected with ice-cold phosphate buffer saline (PBS) pH 7.4. Cell pellets were resuspended in ice-cold 0.1% NP40 (Calbiochem), and then used as “whole cell lysate.” Laemmli sample buffer (Bio-Rad) was added into part of the whole cell lysate, and then these lysates were sonicated and centrifuged. The supernatant was used as the “cytosolic fraction.” To extract the “nuclear fraction,” the remaining pellets were resuspended and boiled with Laemmli sample buffer, and then centrifuged to remove the supernatant. The pellets were resuspended again with ice-cold 0.1% NP40 in PBS, centrifuged to remove the supernatant, and resuspended with Laemmli sample buffer. This was used as “nuclear fraction.” “Whole cell lysate,” “cytosolic fraction,” and “nuclear fraction” were then subjected to Western blotting to determine the location of specific proteins.

### Western Blotting Analysis

Samples for Western blotting were lysed in RIPA buffer (50 mM Tris-HCl pH8.0, 150mM NaCl, 1 mM EDTA pH 8.0, 1 mM EGTA pH 8.0, 0.1% SDS, 0.5% deoxycholate, 1% Triton) and extracted using a sonicator (Qsonica). Protein concentration was measured and quantitated using the Bradford assay (Pierce). Sodium dodecyl sulfate polyacrylamide gel electrophoresis (Bio-Rad) was performed to separate crude proteins, and then proteins were transferred onto a PVDF membrane (Bio-Rad) using protein mini trans-blot cells (Bio-Rad). For the Western blotting, PVDF membranes were blocked in 5% skimmed milk, and then incubated with the primary antibodies, including ATXN3 (GeneTex GTX 115032; 1:3000 dilution), α-tubulin (GeneTex GTX; 1:10,000 dilution), Histone 3 (Millipore; 1:10,000 dilution), Mfn-1 (Abcam Ab57602; 1:1,000 dilution), Mfn-2 (Abcam Ab124773; 1:2,000 dilution), Drp-1 (Cell signaling NB110-55237; 1:500 dilution), OPA 1 (GeneTex GTX48589; 1:500 dilution), and γ-tubulin (Sigma T6557; 1:10,000 dilution) antibodies. Secondary peroxidase-conjugated antibodies (Jackson ImmunoResearch laboratories) were then applied, and protein expression levels were measured using an Amersham ECL kit (PerkinElmer).

### Immunofluorescence Staining

Neuron 2A or SH-SY5Y cells were cultured and transfected with plasmid DNAs, and then fixed with 4% paraformaldehyde. Fixed cells were blocked with blocking buffer (0.2% triton X-100, sodium azide 3 mM, saponin 0.1%, BSA 2%, donkey serum 5% PBS to final volume) for 1 h and then subjected to hybridization with a flag antibody (Sigma) overnight at 4°C. The next day the primary antibodies were washed out, and these cells were stained with secondary antibodies conjugated with Alexa 488 (Invitrogen). In addition, cellular nuclear were stained with 1 μg/mL Hoechst 33342 (Sigma), and fluorescence images were taken with a DM2500 fluorescent microscope (Leica).

### Cell Survival, Death and Proliferation

The cell survival rate was determined by the 3-(4,5-Dimethylthiazol-2-yl)-2,5-diphenyltetrazolium bromide (MTT) assay. N2a cells transfected with TSQ and TLQ constructs were cultured for 48 h, and MTT was added into the medium with a final concentration of 0.25 mg/mL. Cells were kept culturing for 4 h, the medium was removed and then DMSO was added into culture wells. After mixing for 5 min the optical density was determined by an ELISA reader (BioTek) at the wavelength of 540 nm. Furthermore, the cell death and proliferation rate was analyzed using 0.4% trypan blue dye (Gibco), and dead cells stained by trypan blue dye were calculated via a hemocytometer chamber. In addition, cells not stained by trypan blue dye were also determined using a hemocytometer chamber for cell proliferation.

### Mitochondrial Morphology Analysis

SH-SY5Y cells transfected with TSQ and TLQ constructs were co-transfected with pDsRed2-Mito Vector (Clontech), and then cultured for 48 h. Transfected cells were fixed and fluorescence images were taken with a fluorescent microscope (Leica). DsRed images of mitochondrial morphology were quantitated using the NIH-developed Image J software, and individual mitochondria (particles) were subjected to particle analyses. All mitochondria were quantitated to determine the values for circularity (4p.Area/perimeter^2^), lengths of major/minor axes and the number of particles, and then the form factor (FF, the reciprocal of circularity value) and aspect ratio (AR, major axis/minor axis) were calculated. Higher values of FF and AR indicate an increase in mitochondrial complexity (length and branching) and elongated tubular mitochondria, respectively. Data from different transfected groups were subjected to statistical analyses.

### TMRE-Mitochondrial Membrane Potential Assay

Mitochondrial membrane potential (MMP) was assayed by Tetramethylrhodamine, ethyl ester, perchlorate (TMRE; Invitrogen)-MMP assay. Briefly, N2a cells were cultured and transfected in a 96-well plate for 48 h, and then the culture medium was removed. 250 nM of TMRE staining solution was added into each well, and then incubated in a CO_2_ incubator at 37°C for 1 h. The staining solution was discarded, and washed and replaced with 1xPBS. The fluorescent intensities of the TMRE were determined using an ELISA reader (BioTek) with the setting Ex/Em = 530/595 nm. As to brain tissues, mice were anesthetized and perfused using 4% paraformaldehyde (Sigma), and brain samples were fixed in 4% paraformaldehyde. Post-mortem brains were then cryosectioned with 25 μm thickness, and then subjected into 25 nM TMRE staining solution for 1 h. The staining solution was discarded, and washed and replaced with 1xPBS. The fluorescent images of the TMRE were examined by a DM2500 fluorescent microscope (Leica) and Leica Application Suite software (Leica).

### Measurement of Reactive Oxygen Species (ROS)

Reactive oxygen species in transfected cells was determined by the measurement of 2′,7′-dichlorofluorescindiacetate (DCFDA) and 2′,7′-dichlorofluorescein (DCF) using Reactive Oxygen Species Detection Reagents (Invitrogen). N2a cells transfected with TSQ and TLQ constructs were cultured for 48 h, washed with PBS, and then mixed with PBS containing 5 μg/μL DCFH-DA. DCFDA was deacetylated and then oxidized by ROS into DCF, and fluorescent signals of DCF were determined via 485 nm excitation and 530 nm emission wavelength using an ELISA reader (BioTek).

### Immunohistochemical (IHC) Staining for Mouse Brain

Mice were anesthetized and perfused using 4% paraformaldehyde (Sigma), and brain samples were fixed in 4% paraformaldehyde. Post-mortem brains were then cryosectioned with 25 μm thickness and these sections were incubated with 0.3% hydrogen peroxide for 15 min, blocked for 1 h at room temperature, and incubated with the ATXN3 primary antibody (GeneTex) at 4°C overnight. After washing with DPBS, the brain sections were processed using Vectastain Elite ABC kit (Vector Laboratories), and 3,3′-diaminobenzidine (DAB, Vector Laboratories), and then mounted on the slides with mounting media (Ted Pella). Images were examined by a DM2500 microscope (Leica) and captured by Leica Application Suite software (Leica).

### Fluoro-Jade B Staining

The neurodegeneration was determined by Fluoro-Jade B staining (Millipore) following the protocol provided by the manufacturer. Briefly, the cerebellums of mice were fixed with 4% paraformaldehyde, and then subjected to frozen sectioning. The cerebellum sections were incubated with 1% sodium hydroxide dissolved in 80% alcohol, 70% ethanol and distilled water, consecutively. The sections were transferred to 0.006% potassium permanganate, and then stained with a 0.01% stock Fluoro-Jade B solution. The sections were mounted with mounting media (Ted Pella), and images were examined by a DM2500 microscope (Leica) and Leica Application Suite software (Leica).

### Terminal Deoxynucleotidyl Transferase dUTP Nick End Labeling (TUNEL)

The cell death was determined by *In Situ* Cell Death Detection Kit (Sigma) following the protocol provided by the manufacturer. Briefly, the cerebellum of mice were fixed, cut and mounted on the slides. Then, the sections were incubated in permeabilisation solution (0.1% Triton X-100, 0.1% sodium citrate), washed with PBS, and then resuspended with TUNEL reaction mixture for 1 h at 37°C. After washing with PBS again, the cerebellum sections were covered with mounting media (Ted Pella). Images were examined by a DM2500 microscope (Leica) and captured by Leica Application Suite software (Leica).

### Statistical Analysis

Data were expressed as the mean ± standard deviation. Differences between groups were analyzed using one-way analysis of variance in commercial statistical software (GraphPad Prism 4.02; GraphPad Software, San Diego, CA, United States). Tukey’s procedure was used to test differences among different groups. In some cases Student’s *t*-test was used to compare differences between particular groups. Statistical significance was set at *p* < 0.05.

## Results

### Truncated Mutant ATXN3 Accelerates Nuclear Aggregates *In Vitro*

**FIGURE 2:**
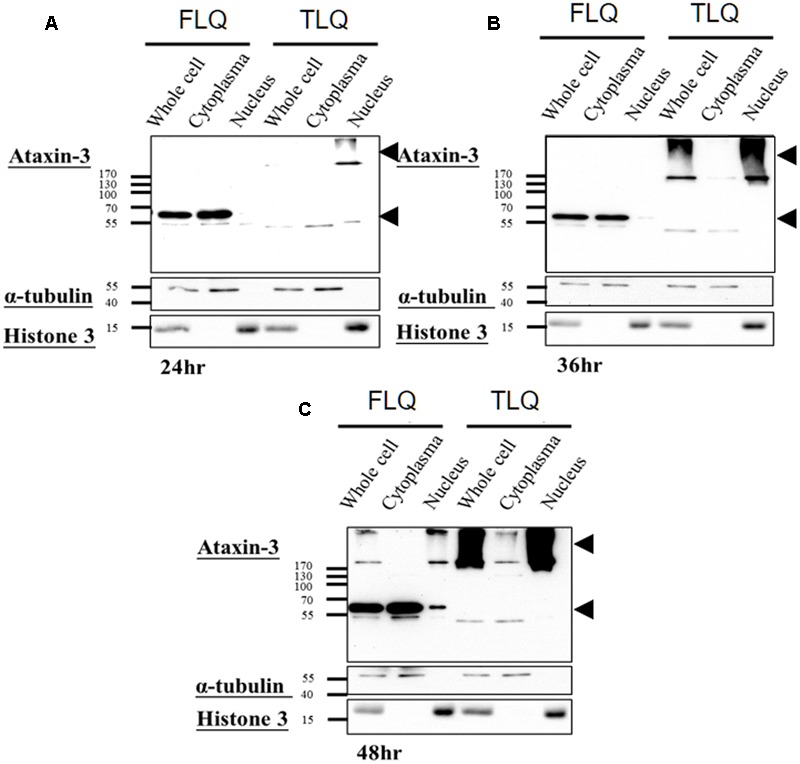
Expression profiling of FLQ and TLQ in cytoplasm and nuclear fractions at different time points. N2a cells were transfected with FLQ or TLQ constructs, and collected for Western blotting using cytoplasm and nuclear fractions 24 **(A)**, 36 **(B)**, and 48 **(C)** h after transfection. The expression of exogenous ATXN3 is indicated by arrow heads. α-tubulin was used as a marker for the cytoplasm fraction, and histone H3 was used as a marker for the nuclear fraction.

The toxic fragment hypothesis of ATXN3 is based on proteolytic cleavage in the ATXN3 protein, accelerating the formation of neuropathological aggregates (Jana and Nukina, 2004; Haacke et al., 2006). However, whether this truncated ATXN3 leads to the dysfunctions of the mitochondria to support the toxic fragment hypothesis is still unknown. Based on the Haacke et al. study (Haacke et al., 2006), when the N-terminus of ATXN3 was deleted more than 186 amino acids, the C-terminus of ATXN3 formed aggregates easier. Since we did not want to observe too severe phenotypes *in vitro* and *in vivo*, we chose the truncated forms of ATXN3, which starts from 163rd amino acid. We first constructed four different ATXN3 expression vectors, including FSQ, FLQ, TSQ, and TLQ (**Figure 1A**). We transfected these four constructs into N2a neuroblastoma cells, and confirmed the expression level of ATXN3 via Western blotting. As shown in **Figure 1B**, we observed soluble forms of FSQ, FLQ, and TSQ at lower molecular weights in separating gel, whereas TLQ formed mutant ATXN3 aggregates at high molecular weight in stacking gel. Furthermore, we also performed immunofluorescence staining, and found N2a cells transfected with TLQ formed intranuclear and periphery aggregates, whereas the FSQ and TSQ displayed a homogenous distribution of ATXN3 signals in cytoplasm (**Supplementary Figure S1**). We also observed N2a cells transfected with FLQ form certain periphery aggregates (**Supplementary Figure S1**). In addition, we collected N2a cells transfected with FLQ and TLQ at 24, 36, and 48 h, and detected the expression of mutant ATXN3 using nuclear and cytoplasmic protein extraction (**Figure 2**). In both constructs, we observed an increase in aggregates with time, especially in the nuclear fraction. However, TLQ led to earlier aggregates in the nucleus than seen with FLQ at 24 or 36 h after transfection (**Figures 2A,B**), and showed much stronger intensity of mutant ATXN3 signals at 48 h after transfection (**Figure 2C**). We also used FSQ and LSQ to transfect N2a cells, and collected cell samples at 48 h for nuclear and cytoplasmic protein extraction and Western blotting, showing that there is no aggregate in both fractions (**Supplementary Figure S2**). These suggest that the truncated form of mutant ATXN3 accelerates the translocation of mutant ATXN3 into the nucleus.

### Truncated Mutant ATXN3 Exacerbates Mitochondrial Morphology and Functions *In Vitro*

**FIGURE 3:**
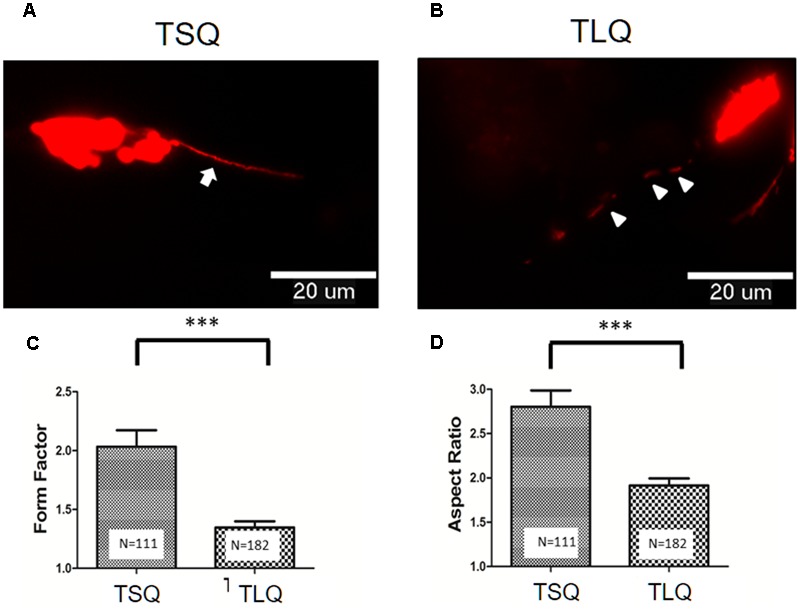
Morphological fusion and fission of mitochondria in SH-SY5Y cells transfected with TSQ and TLQ constructs. SH-SY5Y cells transfected with TSQ **(A)** or TLQ **(B)** were co-transfected with the mitoDsRed, and mitochondrial morphology (Red) in neurites was detected under a florescent microscope. Arrows indicate mitochondrial fusion, and arrow heads indicate mitochondrial fission in neurites. Images were analyzed using the NIH ImageJ software, and statistical quantitation of form factor and aspect ratio are shown in **(C,D)**, respectively. Four independent experiments were examined, and the number of examined mitochondria is indicated inside the bars. ^∗∗∗^ represents *p* < 0.001.

To further address the effects of truncated ATXN3 on mitochondrial functions, we first determined the mitochondrial morphology. TSQ and TLQ constructs were co-transfected with pDsRed2-Mito vectors to trace mitochondrial morphology, such as mitochondrial fusion and fission. As shown in **Figures 3A,B**, we observed TSQ formed continuous and longer mitochondrial fusion, but TLQ showed discontinuous and shorter mitochondrial fission. We further used the ImageJ software to quantitate the mitochondrial fusion and fission, and TLQ had a significantly lower FF and AR than those of TSQ (**Figures 3C,D**), suggesting that TLQ displays relatively more mitochondrial fission status in neuroblastoma cells.

**FIGURE 4:**
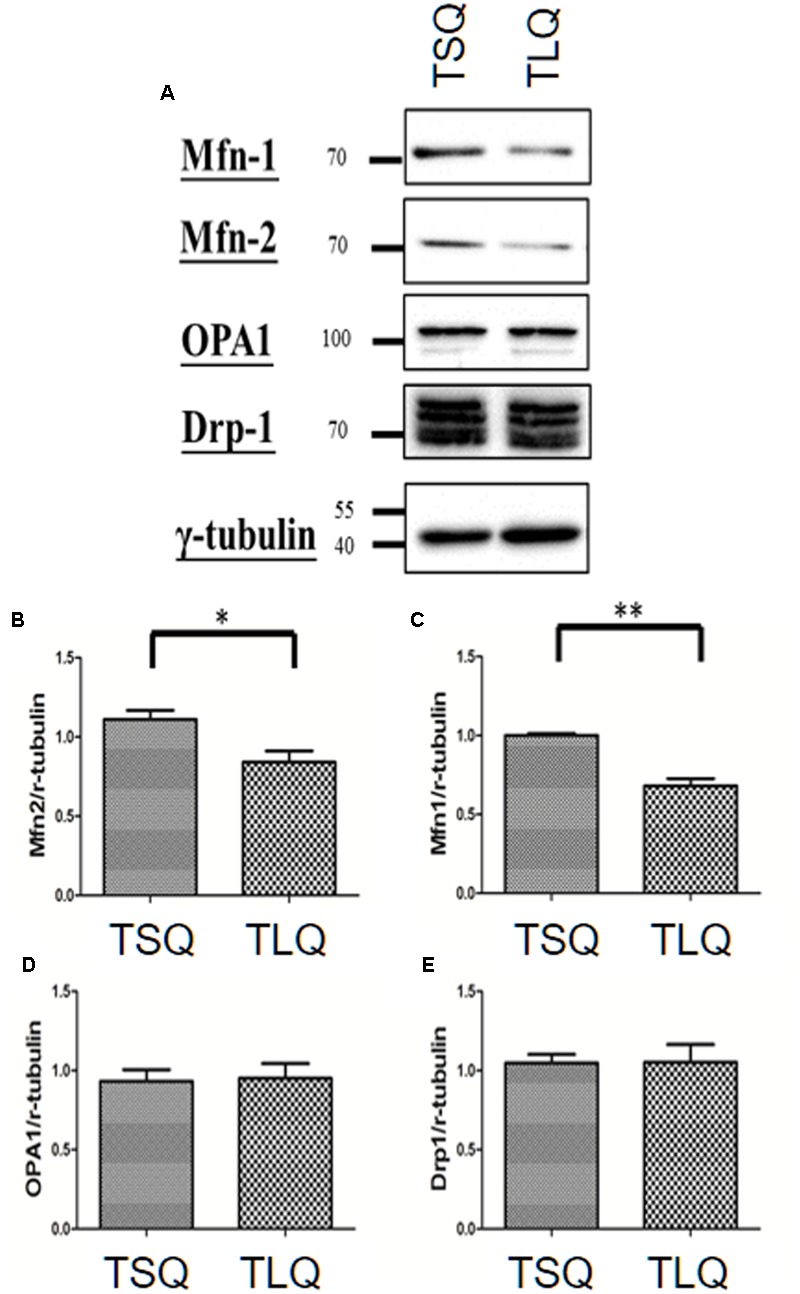
Expression of mitochondrial proteins in TSQ and TLQ N2a cells. N2a cells were transfected with TSQ and TLQ for 48 h, and then subjected to Western blotting using Mfn-1, Mfn-2, OPA1, and Drp-1 antibodies **(A)**. Quantitative results of Mfn-1 **(B)**, Mfn-2 **(C)**, OPA1 **(D)** and Drp-1 **(E)** were normalized by an internal control, γ-tubulin. ^∗^ represents *p* < 0.05 and ^∗∗^ represents *p* < 0.01. Data represent the mean ± SD. *N* = 3 batches of experiments.

There are several critical proteins involved in the mitochondrial dynamics, including Mitofusins 1 (Mfn-1), Mitofusins 2 (Mfn-2), Optic atrophy protein 1 (OPA1), and Dynamin-related protein 1 (Drp-1) (Cho et al., 2010). Mfn-1 and Mfn-2 are two proteins for the fusion of the outer mitochondrial membrane. OPA1 is for the fusion of the inner mitochondrial membrane, and Drp-1 is mediated for mitochondrial fission. We thus further examined the expression profiling of these four proteins in TSQ and TLQ transfected cells (**Figure 4A**), showing Mfn-1 and Mfn-2 proteins were significantly decreased in TLQ cells (**Figures 4A–C**); however, OPA1 and Drp-1 did not show any difference (**Figures 4A,D,E**). We also examine the expression profiling of Mfn-1 and Mfn-2 in N2a cells transfected with FLQ and TLQ, showing TLQ cells display lower trend of Mfn-1 and Mfn-2 expression level (**Supplementary Figure S3**). These results suggest the higher mitochondrial fission status in TLQ cells might be due to lower expression levels of Mfn-1 and Mfn-2.

**FIGURE 5:**
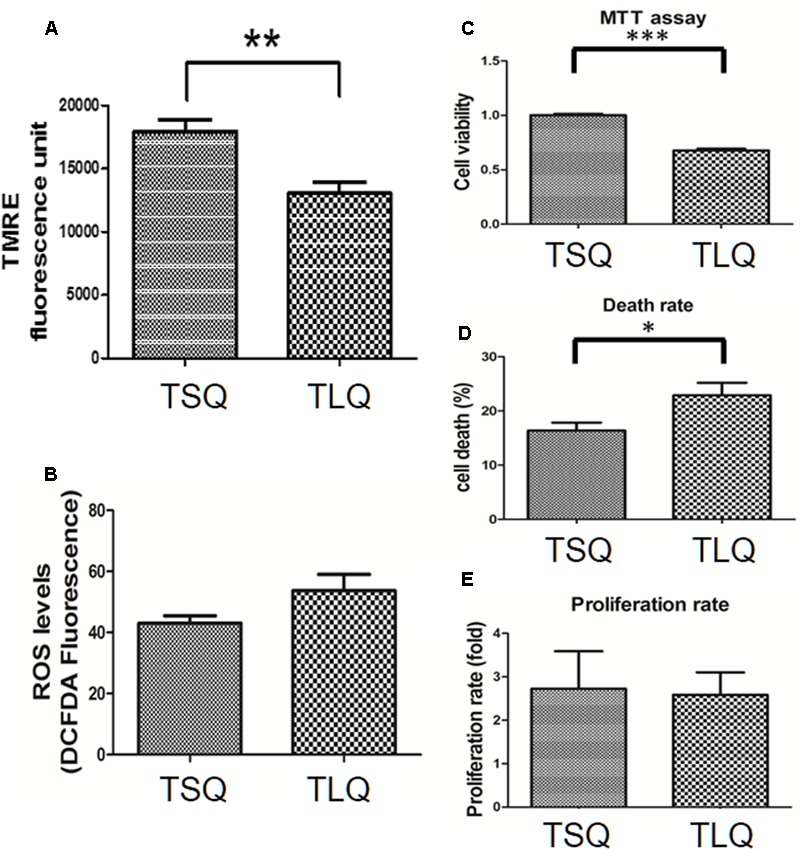
Mitochondrial functions in TSQ and TLQ N2a cells. N2a cell were transfected with TSQ and TLQ for 48 h, and then subjected to examination of mitochondrial membrane potential (MMP) via TMRE-MMP Assay **(A)**, reactive oxygen species (ROS) production via the measurement of 2′,7′-dichlorofluorescein (DCF) **(B)**, cell viability via MTT assay **(C)**, cell death rate **(D)** and cell proliferation **(E)** via trypan blue staining. ^∗^ represents *p* < 0.05, ^∗∗^ represents *p* < 0.01, and ^∗∗∗^ represents *p* < 0.001. Data represent the mean ± SD. *N* = 3 batches of experiments.

Mitochondrial fission morphology might lead to worse functions of the mitochondria, such as a decrease in the MMP and increase in the reactive oxygen species and cell death rate (Cho et al., 2010). To demonstrate on MMP, we transfected N2a cells with TSQ or TLQ, and then subjected these to TMRE-MMP assay. As shown in **Figure 5A**, we observed a significant decrease of TMRE fluorescence signals in the TLQ group, implying TLQ leads to lower MMP. We also compared the MMP among FSQ, TSQ, FLQ, and TLQ cells using the same method, and observed that TLQ significantly decreases the TMRE fluorescence signals compared to those of FSQ, TSQ, and FLQ (**Supplementary Figure S4**). Furthermore, we determined the production levels of ROS, and found TLQ did generate more ROS (**Figure 5B**); however, there was no significant difference between the TSQ and TLQ groups. Since dysfunction of mitochondria finally leads to the death of cells, we further determined the cell survival rate in these two groups. We first used MTT assay to evaluate the cell viability, and TLQ significantly decreased the cell viability compared to TSQ (**Figure 5C**). To further confirm the cell survival rate, we used trypan blue to determine the cell death rate and found TLQ did significantly increase cell death (**Figure 5D**). We also examined the cell proliferation rate after transfection of these two constructs, and the results showed that there was no significant difference between these two groups (**Figure 5E**). The above results suggest that TLQ worsens the functions of the mitochondria and finally leads to death of cells *in vitro*.

### Truncated Mutant ATXN3 Leads to Lower Expression of Mitofusin Proteins and Neurodegeneration *In Vivo*

**FIGURE 6:**
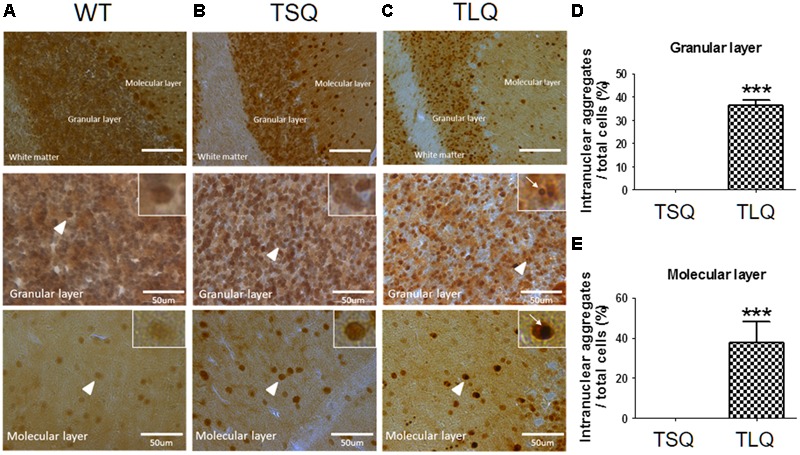
Neuropathological aggregates in cerebellum of truncated ATXN 3 transgenic mice. The cerebellum samples from WT **(A)**, TSQ **(B)**, and TLQ **(C)** mice were collected at 4 months of age, and then subjected to immunohistochemical staining using an ataxin-3 antibody. The upper panel shows molecular layers, granular layers, and white matter under lower magnification. The middle and bottom panels show granular and molecular layers, respectively, under higher magnification. Higher magnification images are shown in upper right squares as the arrow heads indicate. Arrow indicate intranuclear aggregates. Quantitative analyses show the percentages of cells with intranuclear aggregates in granular **(D)** and molecular **(E)** layers. Aggregate numbers are counted from 4 to 6 randomly different 0.02 mm^2^ regions. ^∗∗∗^ represents *p* < 0.001. Data represent the mean ± SD. *N* = 3 for each group.

Since above results were obtained from neuroblastoma cells *in vitro*, we further attempted to examine the dysfunction of mitochondria using TSQ and TLQ transgenes *in vivo*. We generated TSQ and TLQ transgenic mice via lentiviral transgenesis (Cheng et al., 2016), and confirmed genotyping of these founders using PCR (**Supplementary Figures S5A,B**). We also extracted raw protein from the cerebellums of these mice at 4–6 months of age, and confirmed the expression of mutant ATXN3 via Western blotting (**Supplementary Figure S5C**). In TLQ transgenic mice, we found mutant ATXN3 formed much more aggregates in stacking gels compared to those of the same aged controls (**Supplementary Figure S5C**). However, we did not observe significantly abnormal appearance and motor ability at this stage compared to non-transgenic littermates. In addition, immunohistostaining using an ATXN3 antibody was performed in the cerebellums of these mice at 4 months of age (**Figure 6**). ATXN3 signaling was detected in the granular and molecular layers in wild-type (**Figure 6A**), TSQ (**Figure 6B**), and TLQ (**Figure 6C**) mice, whereas the signal, which was the endogenous ATXN3, was weaker in wild-type mice. In TSQ mice, the signal was homogenously distributed in the nucleus, but there were no intranuclear aggregates; however, the intranuclear aggregates could be easily found in the cerebellums of TLQ transgenic mice. Quantitative results show intranuclear aggregates in granular (**Figure 6D**) and molecular (**Figure 6E**) layers of TLQ transgenic mice are significantly higher than those of TSQ mice. These results suggest TLQ accelerates the formation of intranuclear aggregates in the cerebellum, the most susceptible area of SCA3 patients, *in vivo*.

**FIGURE 7:**
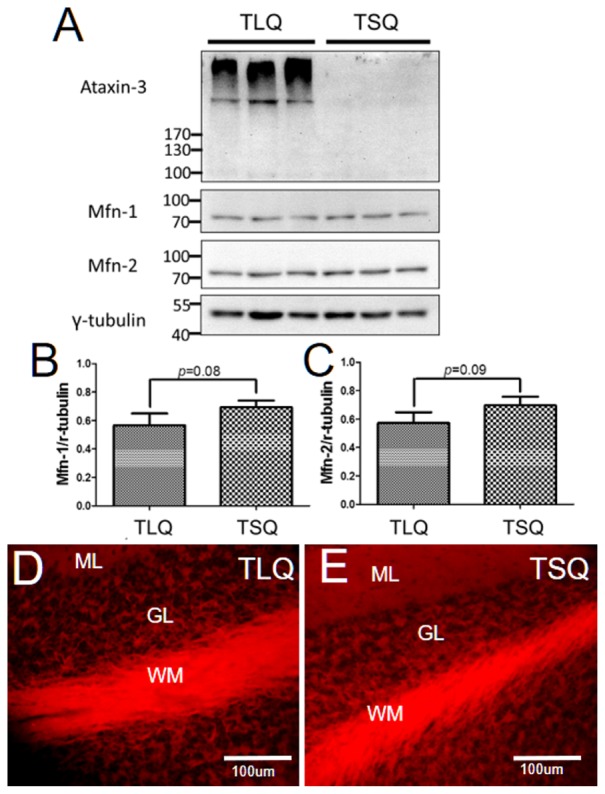
Expression of Mfn-1, Mfn-2, and MMP in cerebellum of TSQ and TLQ transgenic mice. The cerebellum samples from TSQ and TLQ transgenic mice at 4–6 months of age were collected for Western blotting and TMRE assay. **(A)** Western blotting show the expression profiling of ataxin 3, Mfn-1, and Mfn-2. Quantitative results of Mfn-1 **(B)** and Mfn-2 **(C)** were normalized by an internal control, γ-tubulin. MMP functions are shown via TMRE florescence signals in cerebellum of TLQ **(D)** and TSQ **(E)** transgenic mice. Data represent the mean ± SD. *N* = 3 for each group.

Since we detected the significantly lower expression of Mfn-1 and Mfn-2 in the TLQ cells *in vitro* (**Figure 4**), we speculated whether TLQ transgenic mice also show lower expression of these two proteins in cerebellum. We subjected the tissues of cerebellums from TSQ and TLQ transgenic mice to Western blotting using Mfn-1 and Mfn-2 antibodies. As shown in **Figure 7A**, we observed the expression of aggregated ATXN3 at high molecular weight in TLQ transgenic mice. Regarding Mfn-1 and Mfn-2, although there were no significant differences between these two transgenic lines (*p* = 0.08 and *p* = 0.09 for Mfn-1 and Mfn-2, respectively; **Figures 7B,C**), the trends of lower expression in these two proteins in TLQ transgenic mice were observed. We further determine the MMP in cerebellum *in vivo* using TMRE-MMP assay, showing that TLQ transgenic mice have lower TMRE fluorescence signals (**Figure 7D**) compared to those of TSQ transgenic mice (**Figure 7E**), suggesting lower MMP, one of mitochondrial functions, in cerebellum of TLQ mice.

**FIGURE 8:**
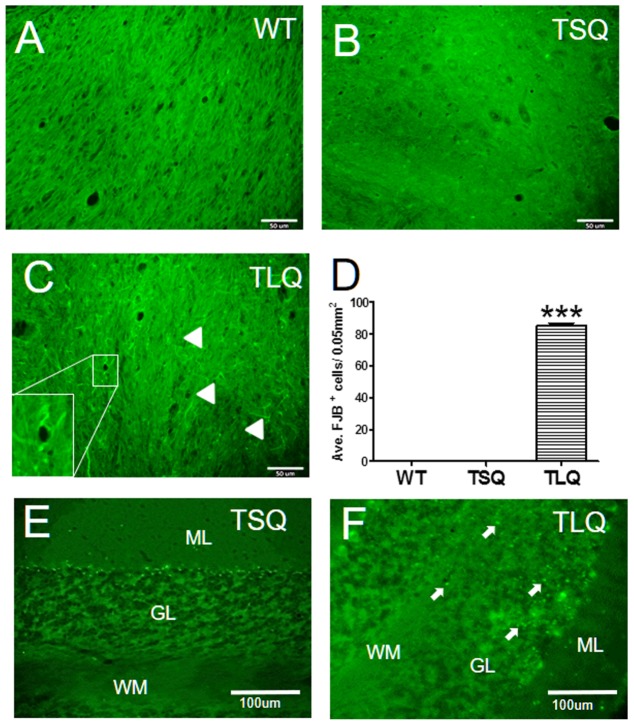
Neurodegeneration and cell death in cerebellum of truncated mutant ATXN 3 transgenic mice. The cerebellum samples from WT **(A)**, TSQ **(B,E)**, and TLQ **(C,F)** mice were collected at 4 months of age, and then subjected to Fluoro-Jade B staining **(A–C)** and TUNEL staining **(E,F)**. Arrow heads in **(C)** indicate degenerative neurons with green filament structure, and higher magnification image of degenerative neurons is shown in bottom left. **(D)** Quantitative results from **(A–C)** are counted from four randomly different 0.05 mm^2^ regions. ^∗∗∗^ represents *p* < 0.001. Arrows in **(F)** show TUNEL-positive dead cells. ML, molecular layer; GL, granular layer; WM, white matter. *N* = 3 for each group.

Finally, since the *in vitro* results showed that TLQ led to dysfunctions of the mitochondria and an increase in cell death, we determined degenerative neurons in these mice using Fluoro-Jade B staining (**Figure 8**). In wild-type and TSQ mice at 4 months of age we did not observe the signal of neurodegeneration in cerebellums (**Figures 8A,B**), whereas positive signals of Fluoro-Jade B staining were detected in TLQ transgenic mice (**Figure 8C**). Quantitation results show that the positive signals of Fluoro-Jade B in cerebellums of TLQ mice are significantly increased compared to those in wild-type and TSQ mice (**Figure 8D**). In addition, we also performed terminal deoxynucleotidyl transferase dUTP nick end labeling (TUNEL) to determine dead cells in the cerebellums of TSQ and TLQ mice, showing these are fewer TUNEL positive signals in TSQ mice (**Figure 8E**) compared to those of TLQ mice (**Figure 8F**). These results suggest that TLQ causes more neurodegeneration and cell death in the cerebellums in the transgenic mouse model.

## Discussion

Based on previous studies, the truncated C-terminal fragment of mutant ATXN3 could form cellular aggregates. However, whether this truncated ATXN3 could cause dysfunctions of the mitochondria is still unclear. In this study we observed truncated mutant ATXN3, TLQ, more easily formed insoluble aggregates, and translocated into the nucleus earlier *in vitro*. In addition, this also led to more mitochondrial fission due to lower expression of Mfn-1 and Mfn-2, two proteins for the fusion of the outer mitochondrial membrane. Moreover, TLQ also reduced MMP and generated more ROS, resulting in lower cell survival. In the *in vivo* results, we also found that TLQ transgenic mice displayed more intranuclear aggregates, lower expression of Mfn-1 and Mfn-2, lower MMP and earlier neurodegeneration and cell death. Since we always performed the control experiments using the TSQ construct, these results not only provide solid evidence that truncated C-terminal fragments of mutant ATXN3 disrupt mitochondrial functions *in vitro* and *in vivo*, but also further support the toxic fragment hypothesis in SCA3.

In this study we showed that TLQ accelerates the formation of neuropathological aggregates *in vitro* (**Figures 1, 2** and **Supplementary Figure S1**) and *in vivo* (**Figures 6** and **Supplementary Figure S4**). This result is consistent with a previous report (Haacke et al., 2006), and we also provided *in vivo* evidence of this using transgenic mouse models. These transgenic mouse models will be longitudinally examined with regard to the molecular, pathological and behavioral phenotypes. We expect these models will provide not only evidence to support the toxic fragment hypothesis in SCA3, but also a truncated ATXN3 mouse model, which may reduce the window of disease progression for the field of SCA3.

Mitochondrial deficit is often reported in polyglutamine diseases, such as spinal and bulbar muscular atrophy and Huntington’s disease (Yano et al., 2014; Borgia et al., 2017). The dysfunction of the mitochondria, including increase of mitochondrial-mediated cell death, decrease of mitochondrial DNA copy numbers and disturbance of mitochondrial complex II, has previously been reported in SCA3 cells, transgenic mice and patients. (Tsai et al., 2004; Chou et al., 2006; Yu et al., 2009; Laco et al., 2012; Kazachkova et al., 2013). However, these studies are all based on the model of full-length mutant ATXN3. Our truncated ATXN3 model showed abnormal morphology and dysfunction of mitochondria, and finally led to neurodegeneration or cell death *in vitro* and *in vivo* (**Figures 3–5, 7, 8**). These results are quite comparable to the studies of full-length mutant ATXN3 models, suggesting that truncated C-terminal ATXN3 is a critical component leading to mitochondrial dysfunction in SCA3.

Based on previous reviews, mitochondrial morphology, including fusion and fission, affects in mitochondrial integrity and functions (Bertholet et al., 2016; Guedes-Dias et al., 2016). Generally, mitochondrial fission leads to the production of small mitochondrial fragments, resulting in lower MMP. In several disease models, such as Parkinson’s or Huntington’s diseases, relatively more mitochondrial fissions are observed in diseased neuronal cells (Manczak and Reddy, 2015; Peng et al., 2016). Furthermore, our truncated ATXN3 model also revealed more mitochondrial fissions (**Figure 3**), suggesting that mitochondrial fission is a common characteristic of neurodegenerative diseases. To the best of our knowledge, our results are not only the first to show the abnormal mitochondrial fusion/fission in SCA3 models, but also imply that therapies targeting mitochondrial dynamics/functions might be an important direction for these neurodegenerative diseases.

Mfn-1, Mfn-2, OPA1, and Drp-1 are important molecules regulating mitochondrial dynamics (Cho et al., 2010; Bertholet et al., 2016). Generally, Mfn-1, Mfn-2, and OPA1 are responsible for mitochondrial fusion, whereas Drp-1 is the major mediator for mitochondrial fission. In this study, truncated ATXN3 led to more mitochondrial fission (**Figure 3**), and decreased levels of Mfn-1 and Mfn-2, not OPA1 and Drp-1, were observed (**Figure 4**). In the *in vivo* results, we also observed lower expression trends of these two proteins in the cerebellum of TLQ transgenic mice, although the difference was not significant (**Figure 7**). We speculate that these mice might not be old enough for severer expression profiling. However, we did observe neurodegeneration in the TLQ transgenic mice (**Figure 8**). Therefore, combining the *in vitro* and *in vivo* results suggests that Mfn-1 and Mfn-2 might play more important roles in mitochondrial dynamics during the progression of SCA3. Mutant ATXN3 has been reported to sequester other interacting proteins into aggregates, and causes the dysfunctions of these interacting proteins (Donaldson et al., 2003; Yang et al., 2014). Therefore, whether our truncated ATXN3 sequesters Mfn-1 and Mfn-2 to disrupt their functions is an issue that still needs further investigation.

Taken together, this study provides evidence showing that the truncated ATXN3 accelerates the formation of aggregates, disrupts the dynamics of mitochondria, and leads to neuronal death *in vitro* and *in vivo*. These results support not only the toxic fragment hypothesis in SCA3, but also the critical roles of truncated ATXN3 for mitochondrial dysfunctions, with further implications for therapeutic developments with regard to mitochondria in SCA3.

## Author Contributions

Y-LJ, P-HC, Y-FC, S-HM, H-IY, C-WL, and S-HY handled animal studies, molecular analysis, and analyzed data; J-YH, C-MC, and S-HY designed the experiments and oversaw the progression of this study. J-YH, C-MC and S-HY drafted the paper. All authors read and approved the final manuscript.

## Conflict of Interest Statement

The authors declare that the research was conducted in the absence of any commercial or financial relationships that could be construed as a potential conflict of interest.

## Supplementary Material

FIGURE S1 The distribution of exogenous ATXN3 in FSQ, FLQ, TSQ, and TLQ N2a cells. N2a cells were transfected with FSQ **(A)**, FLQ **(B)**, TSQ **(C)** or TLQ **(D)**, and then subjected to immunofluorescence staining using a flag antibody (Green). Hoechst staining (blue) shows the location of the nuclei. Arrows indicate the expression of exogenous ATXN3 homogeneously, and arrow heads indicate aggregates of ATXN3.

FIGURE S2 Expression profiling of ATXN3 after FSQ, FLQ, TSQ or TLQ overexpression in cytoplasm and nuclear fractions. N2a cells were transfected with FSQ (**A**, left panel), FLQ (**A**, right panel), TSQ (**B**, left panel) or TLQ (**B**, right panel) constructs, and collected for Western blotting using cytoplasm and nuclear fractions 48 h after transfection. Ataxin-3 was used as a marker for ATXN3 expression, α-tubulin was used as a marker for the cytoplasm fraction and histone H3 was used as a marker for the nuclear fraction.

FIGURE S3 The expression profiling of Mfn-1 and Mfn-2 in FLQ and TLQ N2a cells. N2a cells were transfected with FLQ and TLQ for 48 h, and then subjected to Western blotting using Mfn-1 **(A)** and Mfn-2 **(B)** antibodies. The expression levels were normalized by an internal control, γ-tubulin, and showed in the bottom of **(A,B)**.

FIGURE S4 Mitochondrial membrane potential in FSQ, TSQ, FLQ, and TLQ N2a cells. N2a cell were transfected with FSQ, TSQ, FLQ or TLQ for 48 h, and then subjected to examination of MMP via TMRE-Mitochondrial Membrane Potential Assay. Different characters on different bars indicate statistically significant difference. Data represent the mean ± SD. *N* = 3 for each group.

FIGURE S5 Generation of TSQ and TLQ transgenic mice. Transgenic mice were generated via lentiviral transgenesis. Transgenic status was confirmed by PCR in TSQ **(A)** and TLQ **(B)** transgenic mice. Wild-type mice (WT) were used as the negative control, and 293 FT cells transfected with plasmid DNAs were used as the positive control (PC). The expression of cerebellum ATXN3 in different mice was examined by Western blotting using an ataxin-3 antibody **(C)**. Different lanes present different individual transgenic mice as indicated. Cells transfected with TSQ (TSQ PC) and TLQ (TLQ PC) were used as positive controls. The expression of exogenous ATXN3 is indicated by arrow heads. β-actin was used as an internal control.

## References

[B1] Bertholet A. M. Delerue T. Millet A. M. Moulis M. F. David C. Daloyau M. (2016). Mitochondrial fusion/fission dynamics in neurodegeneration and neuronal plasticity. *Neurobiol. Dis.* 90 3–19. 10.1016/j.nbd.2015.10.011 26494254

[B2] Borgia D. Malena A. Spinazzi M. Desbats M. A. Salviati L. Russell A. P. (2017). Increased mitophagy in the skeletal muscle of spinal and bulbar muscular atrophy patients. *Hum. Mol. Genet.* 26 1087–1103. 10.1093/hmg/ddx019 28087734 PMC5409076

[B3] Chen C. M. Weng Y. T. Chen W. L. Lin T. H. Chao C. Y. Lin C. H. (2014). Aqueous extract of Glycyrrhiza inflata inhibits aggregation by upregulating PPARGC1A and NFE2L2-ARE pathways in cell models of spinocerebellar ataxia 3. *Free Radic. Biol. Med.* 71 339–350. 10.1016/j.freeradbiomed.2014.03.023 24675225

[B4] Cheng P. H. Chang Y. F. Mao S. H. Lin H. L. Chen C. M. Yang S. H. (2016). Lentiviral transgenesis in mice via a simple method of viral concentration. *Theriogenology* 86 1427–1435. 10.1016/j.theriogenology.2016.04.088 27264740

[B5] Cho D. H. Nakamura T. Lipton S. A. (2010). Mitochondrial dynamics in cell death and neurodegeneration. *Cell Mol. Life. Sci.* 67 3435–3447. 10.1007/s00018-010-0435-2 20577776 PMC11115814

[B6] Chou A. H. Chen Y. L. Hu S. H. Chang Y. M. Wang H. L. (2014). Polyglutamine-expanded ataxin-3 impairs long-term depression in Purkinje neurons of SCA3 transgenic mouse by inhibiting HAT and impairing histone acetylation. *Brain Res.* 1583 220–229. 10.1016/j.brainres.2014.08.019 25139423

[B7] Chou A. H. Yeh T. H. Kuo Y. L. Kao Y. C. Jou M. J. Hsu C. Y. (2006). Polyglutamine-expanded ataxin-3 activates mitochondrial apoptotic pathway by upregulating Bax and downregulating Bcl-xL. *Neurobiol. Dis.* 21 333–345. 10.1016/j.nbd.2005.07.011 16112867

[B8] Donaldson K. M. Li W. Ching K. A. Batalov S. Tsai C. C. Joazeiro C. A. (2003). Ubiquitin-mediated sequestration of normal cellular proteins into polyglutamine aggregates. *Proc. Natl. Acad. Sci. U.S.A.* 100 8892–8897. 10.1073/pnas.1530212100 12857950 PMC166409

[B9] Guedes-Dias P. Pinho B. R. Soares T. R. De Proenca J. Duchen M. R. Oliveira J. M. (2016). Mitochondrial dynamics and quality control in Huntington’s disease. *Neurobiol. Dis.* 90 51–57. 10.1016/j.nbd.2015.09.008 26388396

[B10] Haacke A. Broadley S. A. Boteva R. Tzvetkov N. Hartl F. U. Breuer P. (2006). Proteolytic cleavage of polyglutamine-expanded ataxin-3 is critical for aggregation and sequestration of non-expanded ataxin-3. *Hum. Mol. Genet.* 15 555–568. 10.1093/hmg/ddi472 16407371

[B11] Jana N. R. Nukina N. (2004). Misfolding promotes the ubiquitination of polyglutamine-expanded ataxin-3, the defective gene product in SCA3/MJD. *Neurotox. Res.* 6 523–533. 10.1007/BF03033448 15639784

[B12] Kawaguchi Y. Okamoto T. Taniwaki M. Aizawa M. Inoue M. Katayama S. (1994). CAG expansions in a novel gene for Machado-Joseph disease at chromosome 14q32.1. *Nat. Genet.* 8 221–228. 10.1038/ng1194-221 7874163

[B13] Kazachkova N. Raposo M. Montiel R. Cymbron T. Bettencourt C. Silva-Fernandes A. (2013). Patterns of mitochondrial DNA damage in blood and brain tissues of a transgenic mouse model of Machado-Joseph disease. *Neurodegener. Dis.* 11 206–214. 10.1159/000339207 22832131

[B14] Laco M. N. Oliveira C. R. Paulson H. L. Rego A. C. (2012). Compromised mitochondrial complex II in models of Machado-Joseph disease. *Biochim. Biophys. Acta* 1822 139–149. 10.1016/j.bbadis.2011.10.010 22037589 PMC3338188

[B15] Manczak M. Reddy P. H. (2015). Mitochondrial division inhibitor 1 protects against mutant huntingtin-induced abnormal mitochondrial dynamics and neuronal damage in Huntington’s disease. *Hum. Mol. Genet.* 24 7308–7325. 10.1093/hmg/ddv429 26464486 PMC4664169

[B16] Matos C. A. De Almeida L. P. Nobrega C. (2016). Proteolytic cleavage of polyglutamine disease-causing proteins: revisiting the toxic fragment hypothesis. *Curr. Pharm. Des.* 10.2174/1381612822666161227121912 [Epub ahead of print]. 28025946

[B17] Neves-Carvalho A. Logarinho E. Freitas A. Duarte-Silva S. Costa Mdo C. Silva-Fernandes A. (2015). Dominant negative effect of polyglutamine expansion perturbs normal function of ataxin-3 in neuronal cells. *Hum. Mol. Genet.* 24 100–117. 10.1093/hmg/ddu422 25143392 PMC4262494

[B18] Paulson H. (2012). Machado-Joseph disease/spinocerebellar ataxia type 3. *Handb. Clin. Neurol.* 103 437–449. 10.1016/B978-0-444-51892-7.00027-9 21827905 PMC3568768

[B19] Paulson H. L. Perez M. K. Trottier Y. Trojanowski J. Q. Subramony S. H. Das S. S. (1997). Intranuclear inclusions of expanded polyglutamine protein in spinocerebellar ataxia type 3. *Neuron* 19 333–344. 10.1016/S0896-6273(00)80943-5 9292723

[B20] Peng K. Yang L. Wang J. Ye F. Dan G. Zhao Y. (2016). The interaction of mitochondrial biogenesis and fission/fusion mediated by PGC-1alpha regulates rotenone-induced dopaminergic neurotoxicity. *Mol. Neurobiol.* 54 3783–3797. 10.1007/s12035-016-9944-9 27271125

[B21] Riess O. Rub U. Pastore A. Bauer P. Schols L. (2008). SCA3: neurological features, pathogenesis and animal models. *Cerebellum* 7 125–137. 10.1007/s12311-008-0013-4 18418689

[B22] Simoes A. T. Goncalves N. Koeppen A. Deglon N. Kugler S. Duarte C. B. (2012). Calpastatin-mediated inhibition of calpains in the mouse brain prevents mutant ataxin 3 proteolysis, nuclear localization and aggregation, relieving Machado-Joseph disease. *Brain* 135 2428–2439. 10.1093/brain/aws177 22843411

[B23] Suzuki K. Bose P. Leong-Quong R. Y. Fujita D. J. Riabowol K. (2010). REAP: a two minute cell fractionation method. *BMC Res. Notes* 3:294 10.1186/1756-0500-3-294 PMC2993727 21067583

[B24] Teixeira-Castro A. Jalles A. Esteves S. Kang S. Da Silva Santos L. Silva-Fernandes A. (2015). Serotonergic signalling suppresses ataxin 3 aggregation and neurotoxicity in animal models of Machado-Joseph disease. *Brain* 138 3221–3237. 10.1093/brain/awv262 26373603 PMC4731417

[B25] Tsai H. F. Tsai H. J. Hsieh M. (2004). Full-length expanded ataxin-3 enhances mitochondrial-mediated cell death and decreases Bcl-2 expression in human neuroblastoma cells. *Biochem. Biophys. Res. Commun.* 324 1274–1282. 10.1016/j.bbrc.2004.09.192 15504352

[B26] Yang H. Li J. J. Liu S. Zhao J. Jiang Y. J. Song A. X. (2014). Aggregation of polyglutamine-expanded ataxin-3 sequesters its specific interacting partners into inclusions: implication in a loss-of-function pathology. *Sci. Rep.* 4:6410 10.1038/srep06410 PMC5377324 25231079

[B27] Yano H. Baranov S. V. Baranova O. V. Kim J. Pan Y. Yablonska S. (2014). Inhibition of mitochondrial protein import by mutant huntingtin. *Nat. Neurosci.* 17 822–831. 10.1038/nn.3721 24836077 PMC4174557

[B28] Yu Y. C. Kuo C. L. Cheng W. L. Liu C. S. Hsieh M. (2009). Decreased antioxidant enzyme activity and increased mitochondrial DNA damage in cellular models of Machado-Joseph disease. *J. Neurosci. Res.* 87 1884–1891. 10.1002/jnr.22011 19185026

