# Supplementary figures and images for: The Truncated C-terminal Fragment of Mutant ATXN3 Disrupts Mitochondria Dynamics in Spinocerebellar Ataxia Type 3 Models
Source: Front Mol Neurosci. 2017 Jun 20;10:196. doi: 10.3389/fnmol.2017.00196 (PMC5476786; doi:10.3389/fnmol.2017.00196)

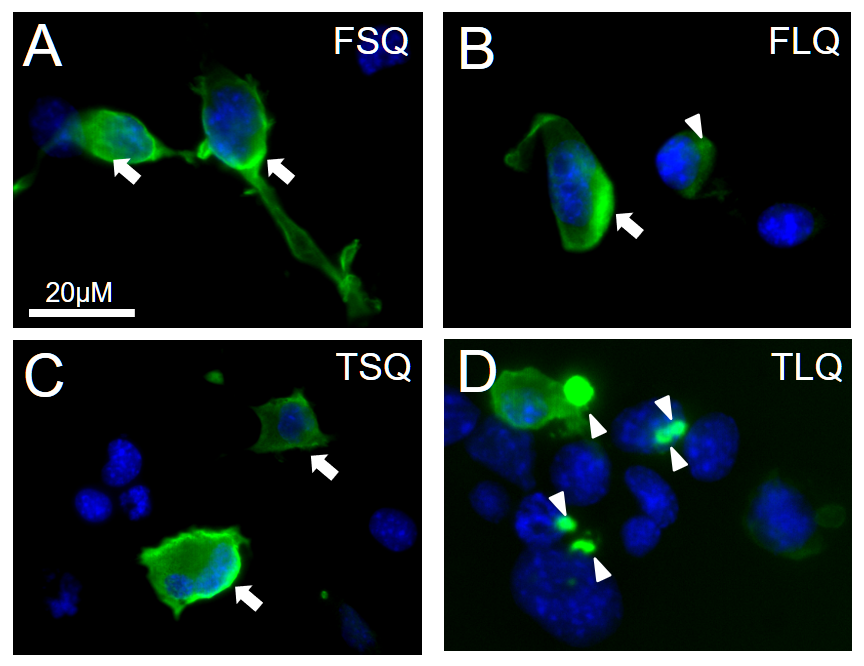

Supplement: FIGURE S1 — The distribution of exogenous ATXN3 in FSQ, FLQ, TSQ, and TLQ N2a cells. N2a cells were transfected with FSQ (A), FLQ (B), TSQ (C) or TLQ (D), and then subjected to immunofluorescence staining using a flag antibody (Green). Hoechst staining (blue) shows the location of the nuclei. Arrows indicate the expression of exogenous ATXN3 homogeneously, and arrow heads indicate aggregates of ATXN3. [file Image_1.tif]

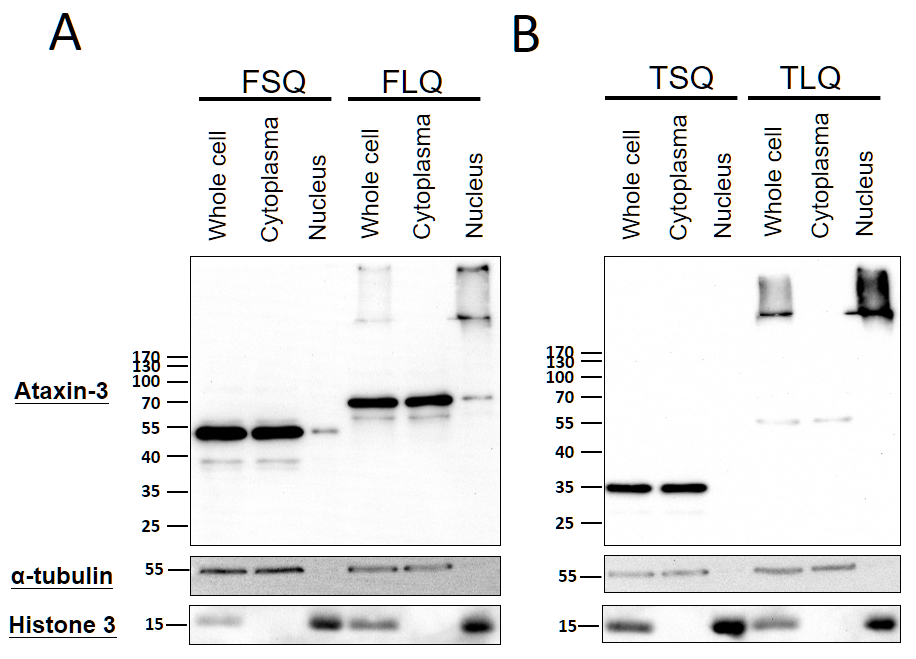

Supplement: FIGURE S2 — Expression profiling of ATXN3 after FSQ, FLQ, TSQ or TLQ overexpression in cytoplasm and nuclear fractions. N2a cells were transfected with FSQ (A, left panel), FLQ (A, right panel), TSQ (B, left panel) or TLQ (B, right panel) constructs, and collected for Western blotting using cytoplasm and nuclear fractions 48 h after transfection. Ataxin-3 was used as a marker for ATXN3 expression, α-tubulin was used as a marker for the cytoplasm fraction and histone H3 was used as a marker for the nuclear fraction. [file Image_2.tif]

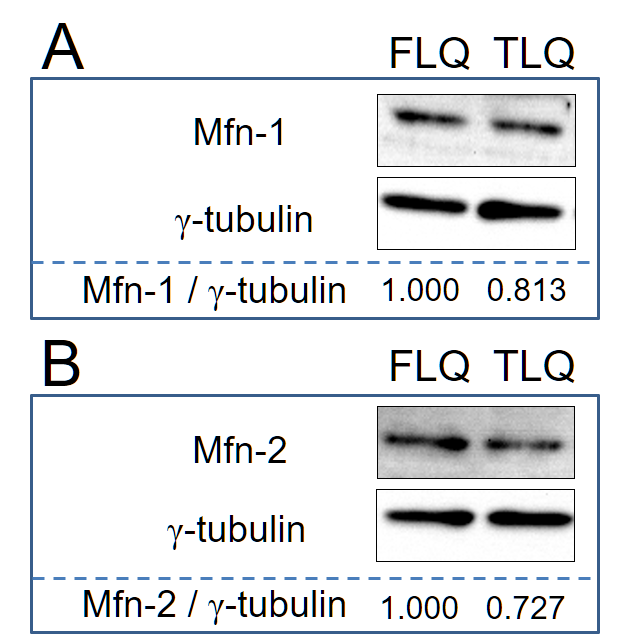

Supplement: FIGURE S3 — The expression profiling of Mfn-1 and Mfn-2 in FLQ and TLQ N2a cells. N2a cells were transfected with FLQ and TLQ for 48 h, and then subjected to Western blotting using Mfn-1 (A) and Mfn-2 (B) antibodies. The expression levels were normalized by an internal control, γ-tubulin, and showed in the bottom of (A,B). [file Image_3.tif]

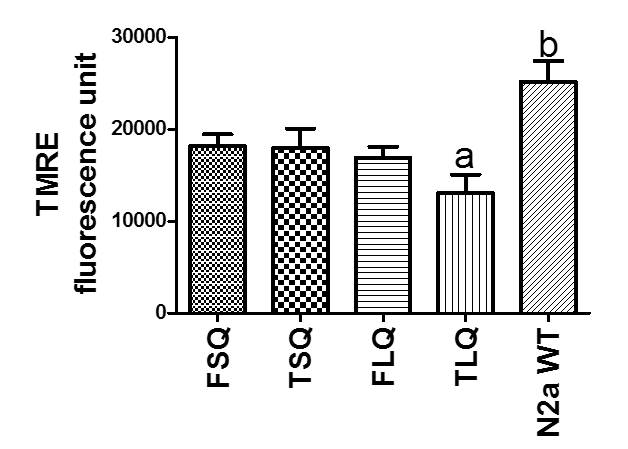

Supplement: FIGURE S4 — Mitochondrial membrane potential in FSQ, TSQ, FLQ, and TLQ N2a cells. N2a cell were transfected with FSQ, TSQ, FLQ or TLQ for 48 h, and then subjected to examination of MMP via TMRE-Mitochondrial Membrane Potential Assay. Different characters on different bars indicate statistically significant difference. Data represent the mean ± SD. N = 3 for each group. [file Image_4.TIF]

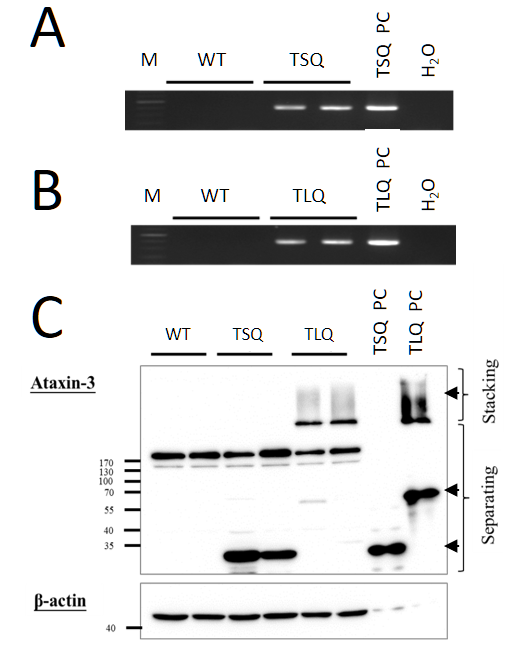

Supplement: FIGURE S5 — Generation of TSQ and TLQ transgenic mice. Transgenic mice were generated via lentiviral transgenesis. Transgenic status was confirmed by PCR in TSQ (A) and TLQ (B) transgenic mice. Wild-type mice (WT) were used as the negative control, and 293 FT cells transfected with plasmid DNAs were used as the positive control (PC). The expression of cerebellum ATXN3 in different mice was examined by Western blotting using an ataxin-3 antibody (C). Different lanes present different individual transgenic mice as indicated. Cells transfected with TSQ (TSQ PC) and TLQ (TLQ PC) were used as positive controls. The expression of exogenous ATXN3 is indicated by arrow heads. β-actin was used as an internal control. [file Image_5.tif]
